# Bioengineered Premna Microphylla-Silver Nanoparticle Hydrogel for Multidrug-Resistant Wound Management in Diabetic Therapeutics

**DOI:** 10.3390/bioengineering13010037

**Published:** 2025-12-29

**Authors:** Pengxiang Xu, Yilong Li, Aidi Tong, Zhou Wu, Chunyi Tong, Bin Liu

**Affiliations:** 1 College of Biology, Hunan Province Key Laboratory of Plant Functional Genomics and Developmental Regulation, Hunan University, Changsha 410082, China; xpx123@hnu.edu.cn (P.X.); li_yilong@peplib.com (Y.L.); tad7223@hnu.edu.cn (A.T.); wuzhou1@hnu.edu.cn (Z.W.); 2 Zonsen PepLib Biotech Inc., Shifeng District, Zhuzhou 412000, China

**Keywords:** plant hydrogel, bacterial infection, diabetic wounds, *Premna microphylla Turcz*

## Abstract

Diabetic wounds are typically difficult to heal. They are usually characterized by prolonged healing time and increased susceptibility to bacterial infection. Therefore, altering the wound microenvironment and improving antibacterial property are effective treatment strategies. In this study, a plant hydrogel with antimicrobial activity and pro-healing properties was designed to integrate silver nanoparticles (AgNPs) with antimicrobial activity into the natural Tofu Chai (*Premna microphylla Turcz*, PMT) hydrogel, which exhibits strong pro-healing ability and antibacterial infections on the wound surface. In vitro experiments showed that AgNPs-PMT had a significant killing effect on Methicillin-resistant *Staphylococcus aureus* (MRSA), with an antibacterial efficiency reaching 95.6%. In vivo results showed that AgNPs-PMT efficiently cleared bacteria at the wound site to promote the formation of neovascularization, collagen and granulation tissue, and facilitated wound healing in a diabetic wound model with MRSA infection. On the 11th day, the wound area was only 5.4% of its original size. Overall, AgNPs-PMT demonstrated favorable antibacterial effects against MRSA and showed great potential in the treatment of chronic diabetic wounds.

## 1. Introduction

The high prevalence of diabetes exists in many countries, often leading to the development of diabetic foot ulcers in late-stage patients [1,2]. Unlike normal acute wounds, diabetic wounds are more difficult to heal [3]. The healing process is significantly hindered by factors such as hyperglycemia, persistent vascular damage, dysregulated inflammatory responses, substantial bacterial invasion, tissue hypoxia, and impaired tissue remodeling [4,5]. Specifically, the abnormal hyperglycemic environment in diabetic patients accelerates bacterial proliferation, leading to the formation of biofilms that enhance drug resistance [6,7]. It also creates a chronic inflammatory environment, damages angiogenesis, and makes wound healing even more challenging [8,9]. Therefore, there is an urgent need for alternative therapeutic approaches to promote diabetic wound healing and treat bacterial infections.

Skin wound healing is a highly coordinated process involving inflammation, re-epithelialization, neovascularization, and extracellular matrix (ECM) remodeling, all of which are essential for effective skin wound repair [10,11]. Among the various biomaterials available today, hydrogels, which have physical properties similar to biological tissues, are considered the most suitable dressing for skin wound healing [12,13]. As a three-dimensional network structure formed by polymer crosslinking, hydrogels exhibit excellent water retention and mechanical properties [14,15]. Thus, they can provide a moist wound environment, simulate the extracellular matrix environment, and act as a drug delivery vehicle, creating an optimal microenvironment for wound healing, thereby promoting the healing of chronic wounds [16,17]. Particularly, hydrogels derived from traditional Chinese herbs, being natural hydrogels, exhibit excellent biocompatibility, biodegradability, and non-toxicity [18,19]. For example, the leaves of *Premna microphylla Turcz* (PMT, a traditional Chinese medicinal herb, other name called Tofu Chai) rich in pectin and various amino acids possess various medicinal properties such as clearing heat, detoxifying, reducing swelling, and stopping bleeding [20,21,22]. Pectin with excellent gel-forming properties can crosslink to form hydrogels in the presence of Ca^2+^ [23,24]. This natural hydrogel with excellent biological safety can serve as an excellent gel carrier to promote wound healing.

As the body’s first line of defense, the skin’s ability to resist bacterial infections is greatly reduced when it is damaged, making it more susceptible to bacterial invasion [25,26]. After bacterial infection, the wound microenvironment undergoes significant changes, including hypoxia, lowered pH, and increased inflammatory factors, which enhance local inflammation and induce oxidative stress, thereby damaging the normal tissue regeneration process around the wound [27,28]. Nanoparticles with a high surface-to-volume ratio have been applied to combat bacterial infections and serve as alternatives to antibiotics, as they are less likely to induce bacterial resistance [29,30]. Among them, silver nanoparticles (AgNPs), a broad-spectrum antimicrobial agent, possess outstanding antibacterial activity by increasing cell membrane permeability, disrupting the cell wall, generating reactive oxygen species (ROS), and ultimately lead to bacterial death [31,32]. However, due to their small size and high surface area, bare AgNPs tend to aggregate easily, significantly reducing their stability and antibacterial effectiveness [33,34]. Moreover, at high concentrations, both AgNPs and the released Ag^+^ ions exhibit considerable cytotoxicity [35,36]. Therefore, a promising strategy is the construction of well-dispersed carriers with excellent biocompatibility to overcome the drawbacks of AgNPs [37,38].

**Scheme 1:**
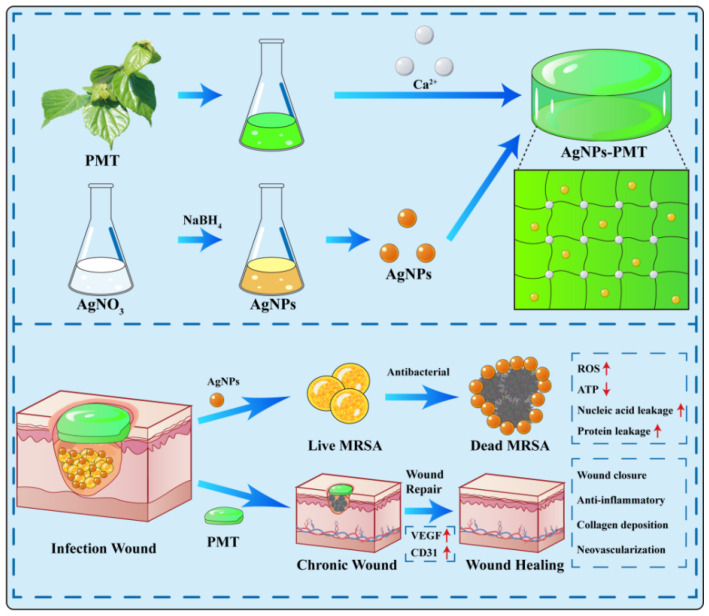
Schematic diagram of the preparation of AgNPs-PMT and their antimicrobial mechanisms in vivo. (Red upward-pointing arrows denote upregulation, while red downward-pointing arrows represent downregulation).

In this study, we used PMT solution and calcium chloride to induce PMT crosslinking through to form hydrogel via Ca^2+^ [39]. Simultaneously, we incorporated AgNPs into the PMT hydrogel by adding an AgNPs solution formed through the reduction of AgNO_3_ by NaBH_4_ [40,41]. The resulting AgNPs-PMT composite hydrogel could release Ag^+^, effectively eradicate Methicillin-resistant *Staphylococcus aureus* (MRSA), and exhibited significant antibacterial activity and good biocompatibility. More importantly, in vivo results demonstrated that in a diabetic mouse MRSA wound infection model, AgNPs-PMT could degrade and eliminate bacteria at the wound site, significantly promoting neovascularization, collagen formation, and granulation tissue development, ultimately accelerating wound healing (Scheme 1).

## 2. Materials and Methods

### 2.1. Materials

*Premna microphylla Turcz* (PMT) leaves were obtained from the local market (Chongqing, China). Silver nitrate (AgNO_3_), Trisodium citrate (C_6_H_5_Na_3_O_7_), Calcium chloride dihydrate (CaCl_2_·2H_2_O) were purchased from Sinopharm Chemical Reagent Co., Ltd. (Shanghai, China), Sodium borohydride (NaBH4), streptozotocin (STZ) were purchased from Solarbio Technology Co., Ltd. (Shanghai, China).

Fresh PMT leaves were washed with distilled water, followed by blanching in 90 °C hot water for 5 min. The leaves were then rinsed with distilled water, dried in a 50 °C oven, and powdered by grinding. The powder was sieved through a 60-mesh screen to obtain the PMT leaf powder.

### 2.2. Bacterial, Cell Lines and Animals

*MRSA* were obtained from Hunan Provincial Center for Disease Control and Prevention. NIH-3T3 and HUVEC cell lines were obtained from Xiangya Central Laboratory, Central South University. C57BL/6 mice were provided by Hunan Silaike Experimental Animals Co., Ltd. (Changsha, China). The animal experiments were conducted according to the ethical policies and procedures approved by the Institutional Animal Care and Use Committee (IACUC) of College of Biology, Hunan University (2023111201).

### 2.3. Preparation of AgNPs-PMT

A total of 0.2 g of PMT powder was added to 20 mL of deionized water, and 58 mg of calcium chloride dihydrate was added as a crosslinking agent. The mixture was stirred in a 60 °C water bath for 10 min to form a dark green solution, which was then left to stand at room temperature to form a gel. To prepare the AgNPs solution, 1 mL of 0.1 mol/L silver nitrate (AgNO_3_) solution and 1 mL of 0.1 mol/L trisodium citrate solution were added to 100 mL of deionized water. The mixture was stirred at 600 rpm in an ice bath for 30 min. Then, 15 mL of 5 mmol/L freshly prepared sodium borohydride (NaBH_4_) solution was added dropwise, causing the solution to rapidly change from colorless to yellow. The solution was stirred in the ice bath for an additional 30 min, and the final AgNPs solution was stored in a 4 °C refrigerator. To prepare the AgNPs-PMT composite hydrogel, 0.2 g of PMT powder was added to 18 mL of deionized water and stirred in a 60 °C water bath for 5 min. 58 mg of calcium chloride dihydrate was added as a crosslinking agent to form a dark green solution. Then, 2 mL of the AgNPs solution was added, and the mixture was stirred in a 60 °C water bath for 5 min in the dark, forming a deep green solution. The solution was left to stand at room temperature to form a gel.

### 2.4. Characterization

The morphology of the nanoparticles was observed using Transmission Electron Microscopy (TEM) (JEOL, Tokyo, Japan). The morphology of the hydrogels was examined using Scanning Electron Microscopy (SEM) (JEOL, Tokyo, Japan), and the elemental distribution within the hydrogel was determined by Energy Dispersive Spectroscopy (EDS). Rheological tests for the hydrogels were measured by MCR92 advanced rheometer (Anton Paar, Austria) with a 25-mmdiameter parallel plate. Storage modulus (G’) and loss modulus (G”) of hydrogels were monitored with angular frequency sweep from 0.628 to 62.8 rad/s at fixed 1% strain. Moreover, G’ and G” evolutions were obtained under the strain sweep experiments (from 1% to 100%) at a fixed 1 Hz frequency [42]. The chemical structure of the AgNPs-PMT composite was identified by Fourier-Transform Infrared Spectroscopy (FT-IR) using equipment from Puchuan Testing Co., Ltd. (Shenzhen, China). The particle size and zeta potential were determined using the ZetaSizer Nano ZS (Malvern Instruments, Malvern, UK). The characteristic absorption peaks of the nanoparticles were measured using UV/Vis spectroscopy (UV-1800 spectrophotometer, Shimadzu, Kyoto, Japan). The samples (2 mL of PMT, 2 mL of Ag NPs-PMT) were incubated in the 18 mL deionized water at 37 °C, and the released Ag concentration was measured at 0.5, 1, 2, 3, 4, 5, 6, 8, 10, 12, 24, 48, 72, and 96 h of time points by inductively coupled plasma-mass spectrometry (ICP-MS).

### 2.5. In Vitro Cytotoxicity Assay and Hemolysis Assay

Human umbilical vein endothelial cells (HUVECs) and 3T3 cells were incubated at room temperature at a density of 10^4^ cells per well for 1 day. The cells were then incubated with various concentrations of PMT, AgNPs, and AgNPs-PMT for 1 day. Cell viability was measured using the MTT assay. Fresh Balb/c mouse erythrocytes were centrifuged for 5 min at 4 °C and 3500 rpm. The centrifuged erythrocytes were treated with ddH_2_O (as a positive control), PBS (as a negative control), various concentrations of PMT, AgNPs, and AgNPs-PMT. The cytocompatibility of the different samples was determined by centrifuging the samples for photographs and measuring the absorbance intensity of the supernatant with an enzyme marker. The hemolysis rate was calculated according to the following formula:

$$\mathrm{Hemolysis}\;\mathrm{Rate}(\%)=\frac{{\mathrm{OD}}_{\mathrm{test}}-{\mathrm{OD}}_{\mathrm{negative}}}{{\mathrm{OD}}_{\mathrm{positive}}-{\mathrm{OD}}_{\mathrm{negative}}}\times 100\%$$

### 2.6. Cell Migration Assay

The migratory effect of Ag NPs-PMT on Mouse Embryonic Fibroblast (NIH-3T3) was investigated. NIH-3T3 (1 mL, 10^5^ cells/mL) were inoculated in 12-well plates. A straight line was drawn in the middle of the wells with the tip of a 200 μL sterile pipette. The cells were treated with FBS-free medium, PMT, AgNPs (10 μg/mL), and AgNPs-PMT (AgNPs: 10 μg/mL) for 12 h. The migration of the cells was observed with an Olympus IX73 microscope (Evident Corporation, Tokyo, Japan). The scratch area before and after cell migration was simulated with Image J software (1.51j8), and the migration rate was calculated.

$$\mathrm{Migration}(\%)=\frac{A_{0}-A_{t}}{A_{0}}\times 100$$

### 2.7. Bacterial Culture

MRSA was cultured in fresh liquid LB medium obtained from the Hunan Provincial Center for Disease Control and Prevention at 37 °C.

### 2.8. In Vitro Antibacterial Activity

The bactericidal activity of AgNPs-PMT was evaluated by the standard plate count method. Briefly, MRSA was incubated in LB medium at 37 °C for 12 h. Then, 500 μL of PBS, PMT, AgNPs, and AgNPs-PMT were mixed with 500 μL of bacterial culture (OD_600_ = 0.1) and incubated at 37 °C for 2 h. After dilution to 10^5^ CFU/mL, 20 μL of the bacterial culture was inoculated onto LB agar plates and incubated at 37 °C for 24 h. Colony-forming units (CFU) were counted. For the dilution method, bacterial cultures were diluted to an OD_600_ of approximately 0.1. Then, 500 μL of the diluted bacterial suspension was transferred into 1.5 mL centrifuge tubes, with each sample using three tubes. To each tube, 500 μL of PMT, AgNPs, or AgNPs-PMT was added and mixed thoroughly. The tubes were incubated in a 37 °C CO_2_ incubator for 6 h. Every hour, a sample was taken, and the OD600 was measured using a spectrophotometer, with the culture medium used as a blank control. Growth curves were plotted based on the obtained data.

### 2.9. SEM Imaging of MRSA

MRSA treated with PBS, PMT, AgNPs (10 μg/mL), and AgNPs-PMT (AgNPs: 10 μg/mL) was centrifuged at 6000 rpm for 8 min, followed by washing 2–3 times. The bacterial samples were fixed with 2.5% glutaraldehyde at 4 °C for 4 h. After fixation, the samples were dehydrated through a graded series of ethanol solutions for 10 min, and bacterial morphology was observed under a scanning electron microscope (SEM).

### 2.10. ROS Level Testing in MRSA

The ability of MRSA to generate reactive oxygen species (ROS) after treatment with PBS, PMT, AgNPs (10 μg/mL), and AgNPs-PMT (AgNPs: 10 μg/mL) was measured using the DCFH-DA probe. Briefly, MRSA was treated with PBS, PMT, AgNPs (10 μg/mL), and AgNPs-PMT (AgNPs: 10 μg/mL) at 37 °C for 30 min. Then, 10 μM of DCFH-DA probe was added to the treated MRSA and incubated in the dark for 30 min. The fluorescence images of the bacterial samples were obtained using a confocal microscope. To eliminate interference from PMT on the fluorescence intensity readings of ROS, a differential centrifugation protocol was used after antibacterial treatment. First, the mixture was centrifuged at a low speed of 1000 rpm to allow the PMT hydrogel to form a precipitate. The resulting supernatant was then collected and subjected to a higher speed centrifugation at 8000 rpm to harvest the bacterial cells for subsequent fluorescence detection.

### 2.11. Crystal Violet Staining of MRSA Biofilm

MRSA (5 × 10^8^ CFU/mL) was added to a 96-well plate and incubated at 37 °C for 72 h to form a biofilm. After 72 h, the medium was removed from the top of the biofilm. PBS, Vancomycin (Van, 2 μg/mL), PMT, AgNPs (10 μg/mL), and AgNPs-PMT (AgNPs: 10 μg/mL) were added to each well, with the PBS-treated group serving as the negative control. All groups were incubated at 37 °C for 24 h. After fixation with 100 μL methanol, 100 μL of crystal violet solution (dissolved in dimethyl sulfoxide) was added to each well and incubated for 30 min. Excess dye was removed, and the biofilm images were taken.

### 2.12. ATP Level Detection

Bacterial suspension (500 μL with OD_600_ = 0.1) was mixed with 500 μL of each sample, i.e., MRSA (10^8^ CFU/mL) treated with PBS, AgNPs (10 μg/mL), and AgNPs-PMT (AgNPs:10 μg/mL), and incubated at 37 °C for 2 h. The bacterial suspension was centrifuged at 5000 rpm for 5 min at 4 °C, and the supernatant was discarded. The pellet was washed with 1 mL of ddH_2_O, vortexed, and centrifuged again at 5000 rpm for 5 min. The supernatant was discarded, and 500 μL of ATP detection lysis buffer was added to lyse the bacterial cells. The mixture was incubated for 5 min and centrifuged at 12,000 rpm for 5 min at 4 °C. The supernatant was collected and used to measure fluorescence intensity. ATP working solution was prepared by diluting ATP detection reagent with ATP dilution buffer (1:9 ratio). In a 96-well plate, 100 μL of ATP working solution was added to each well, and the plate was incubated at room temperature for 4 min in the dark. Afterward, 20 μL of bacterial supernatant was added to each well, and three parallel replicates were set for each group. Fluorescence intensity was measured using a microplate reader, and relative ATP levels were calculated and analyzed.

### 2.13. Protein and Nucleic Acid Leakage Detection

Bacterial suspension (500 μL with OD_600_ = 0.1) was mixed with 500 μL of each sample, i.e., MRSA (10^8^ CFU/mL) treated with PBS, PMT, AgNPs (10 μg/mL), and AgNPs-PMT (10 μg/mL), and incubated at 37 °C for 2 h. The bacterial suspension was centrifuged at 8000 rpm for 5 min at 4 °C, and the supernatant was collected. The protein content was measured by detecting absorbance at 280 nm using a protein and nucleic acid detector, and relative protein levels were calculated.

Bacterial suspension (500 μL with OD_600_ = 0.1) was mixed with 500 μL of each sample, i.e., MRSA (10^8^ CFU/mL) treated with PBS, PMT, AgNPs (10 μg/mL), and AgNPs-PMT (AgNPs:10 μg/mL), and incubated at 37 °C for 2 h. The bacterial suspension was centrifuged at 8000 rpm for 5 min at 4 °C, and the supernatant was collected. The nucleic acid leakage level was measured by detecting absorbance at 260 nm using a protein and nucleic acid detector.

### 2.14. Wound Healing Assay

Male C57BL/6 mice, aged 6–8 weeks, were obtained from Hunan Sileck Experimental Animal Co., Ltd. To establish the diabetic mouse model, the mice were fed a high-fat diet to reach 25 g in weight. Diabetes was induced by intraperitoneal injection of streptozotocin (STZ, 60 mg/kg), and blood glucose levels were monitored daily. When blood glucose levels were no less than 16.7 mmol/L, the mice were anesthetized, and a 1 cm circular wound was created on the back. Then, 100 μL of MRSA bacterial solution (1 × 10^8^ CFU/mL) was injected into the wound to establish a diabetic infection model. The mice were randomly divided into PBS, PMT, AgNPs (10 μg/mL), and AgNPs-PMT (AgNPs: 10 μg/mL) treatment groups. Each group consisted of five mice. Mouse body weight and wound size changes were recorded daily. Wound areas were quantified using ImageJ (1.51j8) software. After 11 days of treatment, histological analysis of major organs was performed. The skin surrounding the wound was fixed with 4% formaldehyde. After 24 h of fixation, the tissue was dehydrated and defatted using ethanol and xylene. Tissue sections were stained with hematoxylin and eosin (HE) and Masson’s trichrome to assess wound inflammation and collagen regeneration. The major organs of the mice were also stained with HE to evaluate in vivo biocompatibility. Before the biological experiment, the hydrogel was sterilized by exposing it to ultraviolet light for 10 min.

### 2.15. Statistical Analysis

For each experiment, data were obtained from at least three independent experiments and presented as mean results ± standard deviation. Statistical analysis was performed using GraphPad Prism 8 (GraphPad Software, San Diego, CA, USA). Statistical significance was calculated via one-way ANOVA with Tukey’s post hoc test and *t*-test. ns: not significant (*p* > 0.05); * *p* < 0.05, ** *p* < 0.01 *** *p* < 0.001 and **** *p* < 0.0001.

## 3. Results and Discussion

### 3.1. AgNPs-PMT Preparation and Characterization

**Figure 1:**
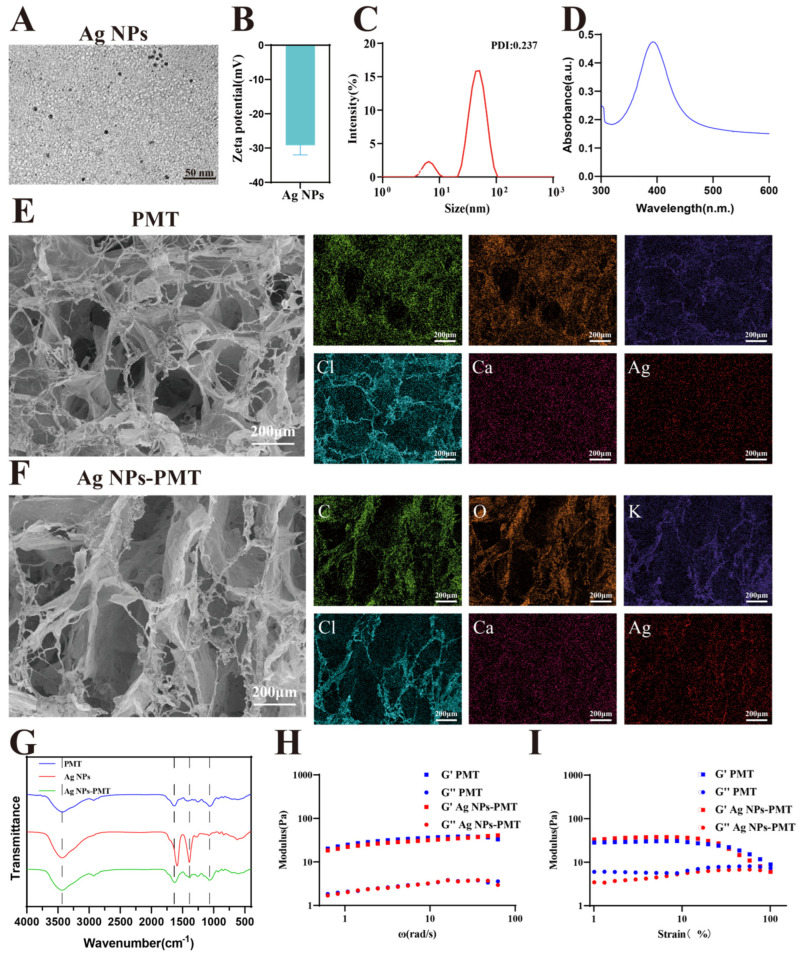
Characterization of PMT, AgNPs and AgNPs-PMT. (**A**) TEM images of AgNPs. (**B**) Zeta potentials of AgNPs. (**C**) The dynamic light scattering (DLS) measurements of AgNPs. (**D**) UV–visible absorption spectra. (**E**) SEM images of PMT and (**F**) AgNPs-PMT. (**G**) FT-IR spectra of PMT, AgNPs and AgNPs-PMT. (**H**) Storage modulus (G’) and loss modulus (G”) of PMT and AgNPs-PMT. Hydrogels, as measured by oscillatory rheology with a frequency sweep. (**I**) Evolution of G’ and G” values for hydrogels under a strain sweep at a fixed frequency of 1 Hz.

In this study, AgNO_3_ was first dispersed in a sodium citrate solution, and NaBH_4_ was used as a reducing agent to synthesize AgNPs. The AgNPs solution was then added to the PMT solution, followed by the addition of CaCl_2_ as a coagulant to form AgNPs-PMT composite hydrogel. Transmission electron microscopy (TEM) images show that the AgNPs have a spherical shape (Figure 1A). Zeta potential measurements reveal that the zeta potential of AgNPs is approximately −29 mV (Figure 1B). Dynamic light scattering (DLS) analysis indicates that the hydrodynamic diameter of the AgNPs is about 62.5 nm (Figure 1C). Figure 1D shows the UV-Vis absorption peak of AgNPs, with an absorption peak observed at 393 nm. The stability of the Ag NPs was evaluated under storage at 37 °C. While the particle size and zeta potential remained largely stable, a gradual increase in the polydispersity index (PDI) was observed, suggesting the onset of localized aggregation (Figure S1).

The prepared PMT and AgNPs-PMT hydrogels were freeze-dried and then subjected to microscopic structural analysis using scanning electron microscopy (SEM). The SEM images revealed that both groups exhibited a three-dimensional network structure (Figure 1E,F). There were no significant changes in the porosity or pore size of the composite hydrogel compared to the blank group. The EDS analysis showed that both hydrogels contained carbon (C), oxygen (O), and potassium (K) from the PMT extract, as well as calcium (Ca) and chlorine (Cl) from the added coagulant CaCl_2_. Additionally, the composite hydrogel contained silver (Ag), which is responsible for its antibacterial activity (Figures S3 and S4). To assess the stability of the hydrogels under application-relevant conditions, the PMT and Ag NPs-PMT hydrogels were stored at 37 °C. After 7 days, both hydrogels retained more than 80% of their initial weight, demonstrating a considerable water retention capacity (Figure S2). The infrared spectrum showed the characteristic absorption bands of the AgNPs-PMT. All samples exhibited strong –OH group IR absorption peaks (3435 cm^−1^). The absorption peaks at 1635 cm^−1^ and 1417 cm^−1^ in PMT resulted from the asymmetric vibration and symmetric vibration of O=C-O in -COO. The absorption peak at 1066 cm^−1^ was caused by the C=O vibration of 3, 6-dehydrated D-galactose. After the incorporation of AgNPs, the characteristic C=O absorption peak originally located at 1635 cm^−1^ underwent a red shift of 11 cm^−1^ to 1624 cm^−1^. This significant shift originates from the coordination interaction between the silver atoms on the surface of AgNPs and the lone pair electrons of the carbonyl oxygen atoms in PMT. The characteristic absorption peak of C-O-C at 1390 cm^−1^ was clearly visible, indicating that AgNPs were successfully loaded on the PMT gel (Figure 1G). To evaluate the drug release capability, the Ag^+^ release profiles of Ag NPs and Ag NPs-PMT were determined by ICP-MS. The Ag NPs exhibited a rapid release, with 51.88% of the Ag^+^ released within the first 0.5 h, approaching completion by 48 h. In contrast, the Ag NPs-PMT showed a significantly slower and more sustained release profile, reaching a cumulative release of 41.12% after 96 h. This controlled release behavior suggests that the PMT matrix effectively modulates the release of silver ions, providing a sustained-release effect (Figure S6).

Moreover, the mechanical properties of the hydrogel need to be carefully considered. The ideal modulus of the hydrogel should be close to that of healthy dermis, providing gentle support for the wound. As we all know, the modulus of dermis ranges from 10 to 50 kPa [43]. Oscillatory rheological measurements show that the storage modulus (G’) values of PMT and Ag NPs-PMT are ~32 Pa and ~40 Pa, respectively, matching that of healthy dermis. These G’ values were significantly higher than the corresponding loss modulus (G”) values (Figure 1H). This G’ > G” relationship indicated the formation of stable polymer networks. Interestingly, the G’ values of both PMT and Ag NPs-PMT decreased as strain increased, as shown by rheological strain sweep tests. When the strain exceeded ~100% for PMT and Ag NPs-PMT, G’ has decreased to be close to below G”, indicating that both hydrogels exhibit shear-thinning properties (Figure 1I). Moreover, time-sweep rheological measurements revealed that both PMT and Ag NPs-PMT exhibited a continuous increase in storage modulus (G’) over time, eventually exceeding the loss modulus (G’’). This indicates that both systems formed gels within 8 min (Figure S5).

### 3.2. In Vitro Antibacterial Activity of AgNPs-PMT

**Figure 2:**
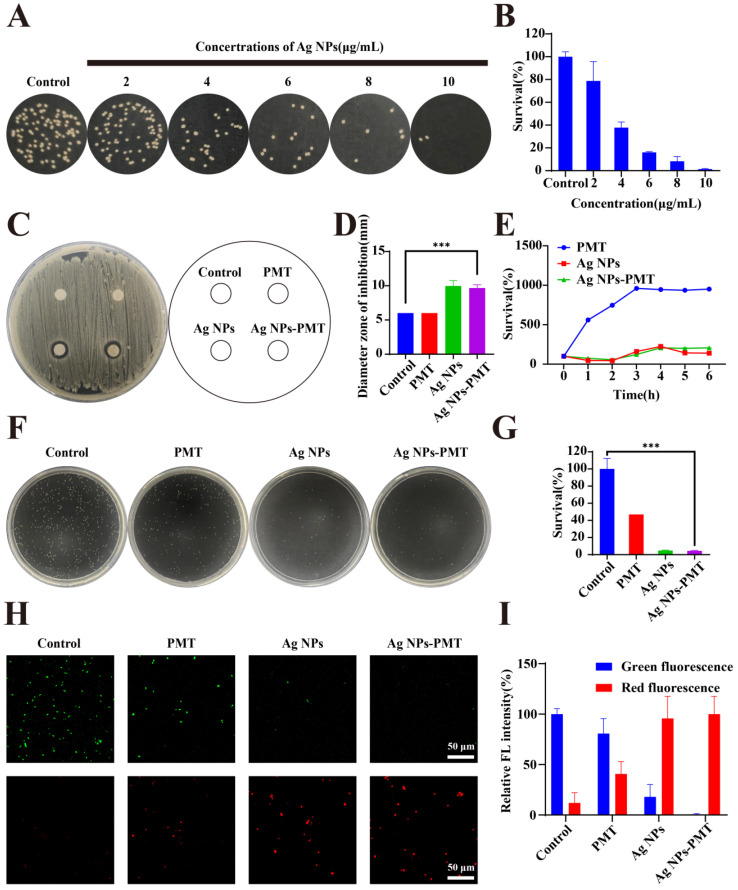
In vitro antibacterial activity. (**A**) Fig. of colonies formed by viable MRSA and (**B**) survival rate of MRSA treated with different concentrations of AgNPs-PMT (AgNPs: 0,2,4,6,8,and 10 μg/mL) were evaluated by CFU counting. (**C**) Photographs and (**D**) the corresponding statistical graphs of inhibition zones of MRSA with different treatments. (**E**) Bacteria-growth curve of MRSA treated with different treatments for 1,2,3,4,5,6 h. (**F**) Photographs of representative plates and (**G**) survival rates of MRSA treatments with PBS, PMT, AgNPs (10 μg/mL), and AgNPs-PMT (AgNPs:10 μg/mL). (**H**) Staining of MRSA with calcein AM/PI dye and observation of bacterial live/dead imaging. (**I**) Quantitative analysis of the relative fluorescence intensity of MRSA. (n = 3, * *p* < 0.05; ** *p* < 0.01; *** *p* < 0.001, **** *p* < 0.0001).

Then, we investigated the in vitro antibacterial activity of the composite hydrogel. The minimum bactericidal concentration (MBC) of AgNPs-PMT against MRSA was evaluated using a plate count method. As shown in Figure 2A,B, AgNPs-PMT showed concentration-dependent bactericidal manner for MRSA in the range from 2 to 10 μg/mL. The MBC of AgNPs-PMT against MRSA was found to be 10 μg/mL, with a bactericidal efficiency reaching 98.6%

Next, the antibacterial effect was estimated by measuring the diameter of the inhibition zone. Consistent with our expectations, PMT had no significant antibacterial effect on MRSA, whereas both AgNPs and AgNPs-PMT exhibited inhibitory effects, as evidenced by distinct inhibition zones of approximately 9.97 mm and 9.67 mm in diameter, respectively (Figure 2C,D). In addition, we investigated the effect of the culture medium on the antibacterial activity of the materials. The bacterial growth curve indicated that both AgNPs and AgNPs-PMT could exert a long-lasting inhibitory effect on MRSA proliferation for up to 6 h in liquid medium (Figure 2E). The plate count assay showed that the antibacterial efficiency of AgNPs and AgNPs-PMT reached 95.2% and 95.6%, respectively (Figure 2F,G). This result demonstrated a slight increase of antibacterial activity in the presence of PMT, likely due to the adsorption and encapsulation of bacteria by the PMT gel, which limited bacterial motility and proliferation, thereby reducing the bacterial content in the bacterial suspension used for plate counting (Figure S7). Additionally, both agar plate and liquid culture results indicated that the composite hydrogel did not significantly impede the release of AgNPs to affect the antibacterial activity. By performing bacterial viability staining to intuitively display fluorescence differences in MRSA after different treatments, we found that the AgNPs and AgNPs-PMT groups showed little green fluorescence, but significant red fluorescence. In contrast, large clusters of green live bacteria were found in the PBS and PMT groups (Figure 2H,I). In conclusion, AgNPs-PMT demonstrated significant antibacterial activity, which primarily stemmed from the antibacterial effect of AgNPs. Furthermore, the incorporation of AgNPs into the composite hydrogel did not interfere with the antibacterial activity of AgNPs.

### 3.3. Exploration of Antibacterial Mechanism

**Figure 3:**
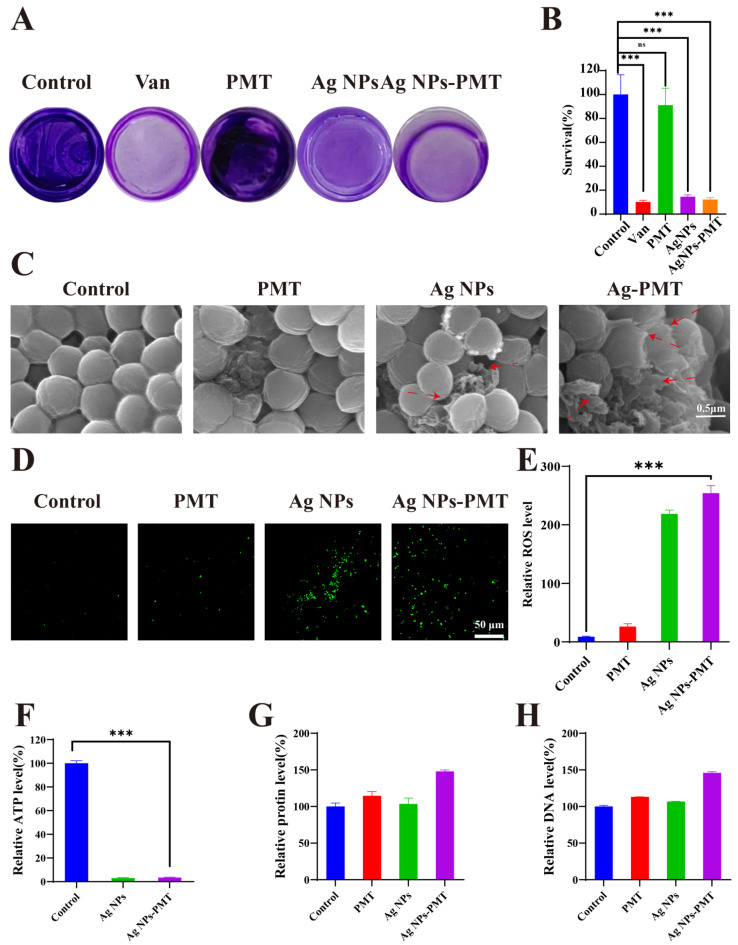
Antibacterial mechanism of AgNPs-PMT. (**A**) Photographs of residual MRSA biofilms after treatment with PBS, Van (2 μg/mL), PMT, AgNPs (10 μg/mL), and AgNPs-PMT (AgNPs: 10 μg/mL). (**B**) MRSA biofilm relative residue assay. (**C**) SEM images of MRSA after treatment with PBS, PMT, AgNPs (10 μg/mL), and AgNPs-PMT (AgNPs:10 μg/mL). Red arrows point to bacteria exhibiting ruptured cell membranes and cell walls. (**D**) Fluorescence images of MRSA and (**E**) plot of relative ROS levels in MRSA. (**F**) ATP level, (**G**) protein leakage and (**H**) DNA leakage of the MRSA suspensions treated with different materials. (n = 3; ns: not significant (*p* > 0.05); * *p* < 0.05; ** *p* < 0.01; *** *p* < 0.001, **** *p* < 0.0001).

It was reported that bacterial resistance mechanisms are primarily based on three lines of defense. The first line of defense is the biofilm formed by bacterial aggregation. Due to the formation of its extracellular matrix (ECM), bacterial clustering, and reduced permeability, biofilms inherently provide microbial protection and resistance, which plays a significant role in wound infections. Considering the strong anti-MRSA effect, we then systematically explored the antibacterial mechanism of AgNPs-PMT. First, we evaluated the biofilm-eradicating ability of AgNPs-PMT. Crystal violet staining results showed that MRSA in the control and PMT groups retained relatively intact biofilm structures, while the Van, AgNPs and AgNPs-PMT groups exhibited significantly lower biofilm content. Quantitative biofilm analysis indicated that AgNPs and AgNPs-PMT reduced biofilm formation by approximately 85.44% and 87.87%, respectively (Figure 3A,B). The biofilm reduction rate achieved by the AgNPs-PMT group (approximately 89.87%) closely approximated that of the vancomycin-positive control group, demonstrating its potent anti-biofilm activity. The second line of bacterial defense is its cell membrane and cell wall. Disruption of the bacterial cell membrane is a common method for killing bacteria. Scanning electron microscopy (SEM) images indicated that MRSA in the PBS and PMT treatment groups appeared spherical, while bacteria treated with AgNPs and AgNPs-PMT showed noticeable surface deformation and damage. The AgNPs-PMT group displayed the most prominent rupture of the cell membrane and cell wall (Figure 3C). Furthermore, protein and nucleic acid assays indicated that, compared to the PBS group, the extracellular nucleic acid levels and extracellular protein concentrations of MRSA treated with PMT and AgNPs were only slightly increased. However, in the AgNPs-PMT-treated MRSA, both extracellular nucleic acid and protein concentrations were significantly elevated (Figure 3G,H), indicating the rupture of bacterial cell membrane caused by AgNPs-PMT. The final line of bacterial defense involves intracellular changes, which are primarily related to metabolic markers. By incubating ROS probe DCFH-DA with MRSA, it was found that no significant green fluorescence was observed in the control and PMT groups, while the strongest green fluorescence signal was detected in the AgNPs and AgNPs-PMT groups (Figure 3D,E). These results suggest that AgNPs-PMT can induce a high level of ROS generation in bacteria. Moreover, the ATP level in MRSA treated with AgNPs and AgNPs-PMT decreased by approximately 96% (Figure 3F). In conclusion, AgNPs-PMT can disrupt biofilm structures, induce bacterial surface deformation, cause rupture of the cell membrane and wall, trigger nucleic acid and protein leakage, increase ROS levels, and reduce ATP levels.

### 3.4. In Vitro Cytocompatibility and Healing Experiments

**Figure 4:**
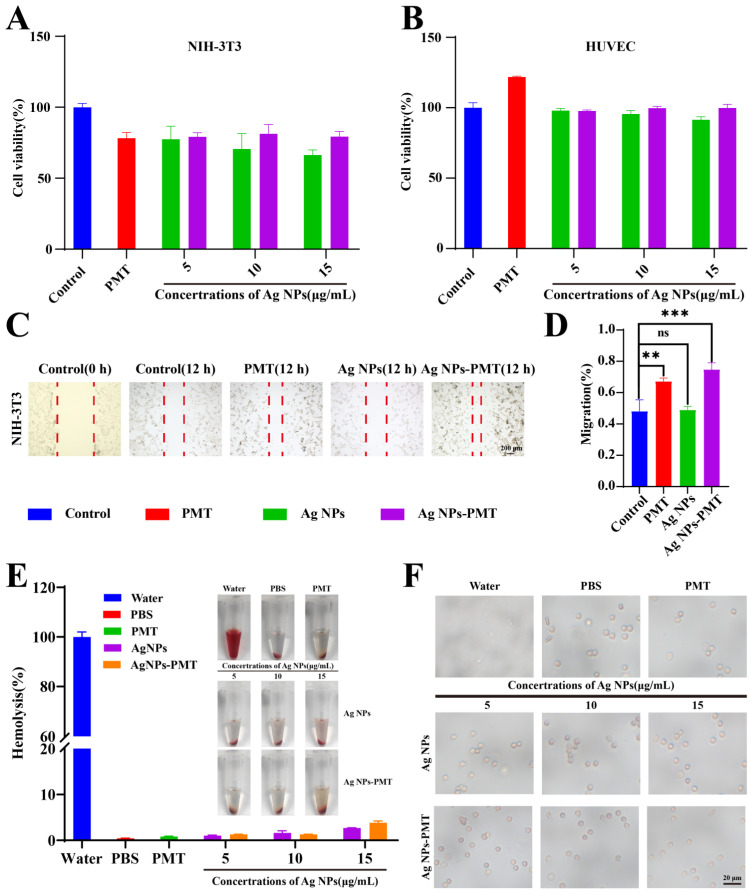
In vitro biosafety of AgNPs-PMT. Viability of (**A**) NIH-3T3 cells and (**B**) HUVEC cells treated with different concentrations of Ag NPs and Ag NPs-PMT for 24 h. (**C**) Digital images of migration of NIH-3t3 cells with different treatments. The red dash line indicated the scratch wound area. (**D**) Quantitative evaluation of cell migration. (**E**) Hemolysis rates and photographs of different concentrations of AgNPs and AgNPs-PMT. (**F**) Morphology of erythrocytes treated with different concentrations of AgNPs and AgNPs-PMT under an inverted microscope. (n = 3; ns: not significant (*p* > 0.05); * *p* < 0.05; ** *p* < 0.01; *** *p* < 0.001, **** *p* < 0.0001,).

Before conducting in vivo experiments, it is crucial to verify the in vitro cytocompatibility of the materials. We evaluated the in vitro cytocompatibility of AgNPs-PMT through cytotoxicity assay and hemolysis test. NIH-3T3 and HUVEC cells were incubated with PMT and different concentrations of AgNPs and AgNPs-PMT for 24 h, followed by cell viability assessment. The results showed that for NIH-3T3 cells, the cell viability in the AgNPs-PMT groups at various concentrations exceeded 80%. For HUVEC cells, the cell survival rate in the AgNPs-PMT group was 99.7%, which was significantly higher than that of 91.4% in the AgNPs group. This result indicated that the PMT gel carrier effectively reduced the cytotoxicity of high concentrations of AgNPs (Figure 4A,B). Next, the effect of AgNPs-PMT on cell migration was evaluated using a scratch assay. Compared with the control group, AgNPs-PMT promoted the highest rate of cell migration (74.79%). Meanwhile, PMT also demonstrated a significant ability to promote cell migration (67.17%). This finding further suggests that AgNPs-PMT can facilitate wound healing primarily by stimulating cell proliferation, a function attributed to PMT (Figure 4C,D). The hemolysis test showed that incubation with AgNPs-PMT resulted in a hemolysis rate of less than 5% and no significant change in red blood cell morphology (Figure 4E,F). In conclusion, AgNPs-PMT exhibited excellent biocompatibility in vitro, providing a promising platform for their potential application.

### 3.5. In Vivo Study of Healing in Diabetic Wounds Infected with MRSA

**Figure 5:**
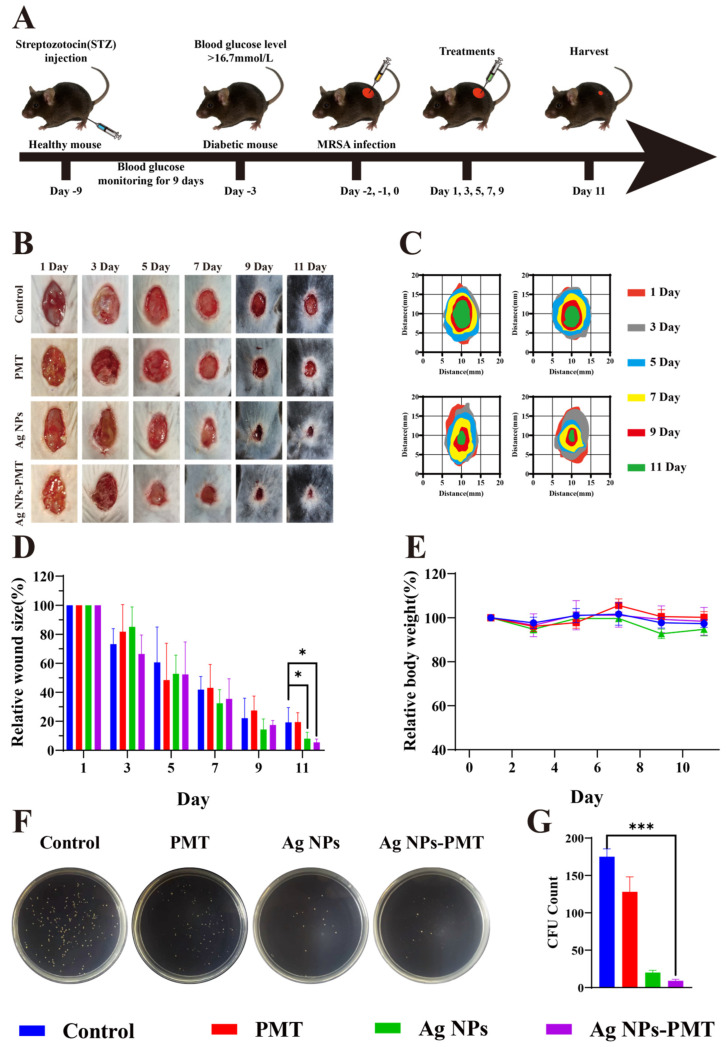
In vivo curative effect of AgNPs-PMT in diabetic mice. (**A**) Schematic representation of MRSA wound infection and AgNPs’ therapeutic profile. Each group consisted of five mice. (**B**) Photographs of the backs of mice after 1, 3, 5, 7, 9, and 11 days of treatment and (**C**) diagram of the wound healing process. (**D**) Wound healing rate of mice during treatment. (**E**) Mice weighed while being treated. (**F**,**G**) Assessment of wound bacterial counts after the fifth day of treatment. (n = 5, * *p* < 0.05; ** *p* < 0.01; *** *p* < 0.001, **** *p* < 0.0001).

Wounds in diabetic patients are more susceptible to microbial infections. Furthermore, the abnormally high blood glucose levels in the wound microenvironment can trigger chronic inflammation and impair angiogenesis, further complicating the healing process. In this study, we evaluated the ability of AgNPs-PMT to eliminate MRSA infection and promote wound healing in diabetic mice. To establish a diabetic mouse model, we induced diabetes by administering streptozotocin (STZ) via continuous intraperitoneal injection for 5 days, which led to the destruction of pancreatic islets. The random blood glucose levels reached 15.8 mM on day 9 post-injection, and the mice’s body weight maintained above 21 g, at which point the wound healing assessment began (Figure S8). A 1 cm diameter circular skin incision was made on the back of the mice, and MRSA was inoculated on the wound surface. The wounds were then treated with PBS, PMT, AgNPs, and AgNPs-PMT (Figure 5A). On the day 11, all groups showed a significant reduction in wound area: the PBS, PMT, AgNPs, and AgNPs-PMT groups reduced the infected wound area to 19.21%, 19.45%, 8.08%, and 5.40%, respectively. The wounds treated with AgNPs-PMT were almost healed by day 11 (Figure 5B–D).

Additionally, compared to the control group, there was no significant change in body weight in the treatment groups, indicating that the treatments did not adversely affect the physiological state of the mice (Figure 5E). To further assess the in vivo antibacterial effect, we compared the bacterial load in the infected wounds on day 5 post-treatment using dilution plating. The results showed that AgNPs-PMT exhibited the highest antibacterial effect among all groups, rapidly and effectively clearing the bacteria, highlighting its strong resistance to bacterial infection and ability to promote wound healing (Figure 5F,G).

**Figure 6:**
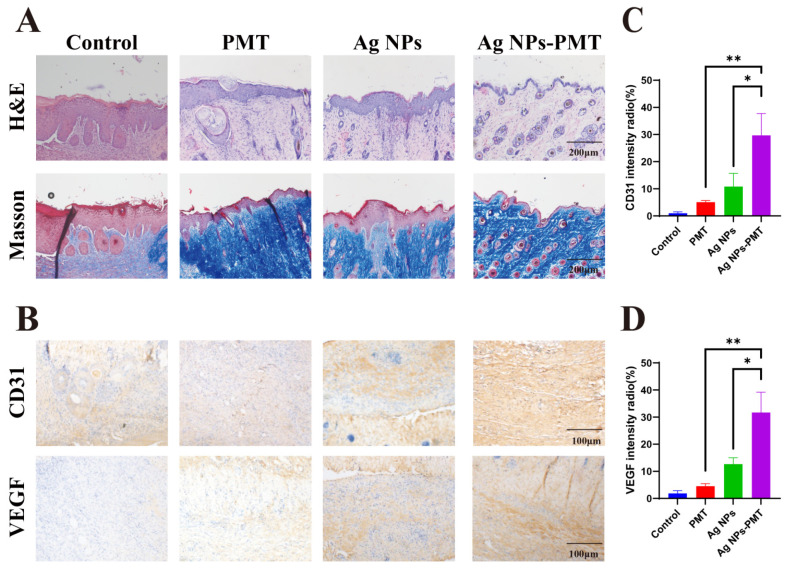
Images of infected wound tissue pathology. (**A**) Images of infected wound sections in H&E and Masson’s staining. (**B**) Images of infected wound sections in CD31 and VEGF’s staining immumohistochemical staining. (**C**) Quantification of CD31 and (**D**) VEGF’s intensity radio. (n = 3, * *p* < 0.05; ** *p* < 0.01; *** *p* < 0.001, **** *p* < 0.0001).

Reduction of inflammation and promotion of angiogenesis play crucial roles in diabetic wound healing. To evaluate the healing process of wounds, we conducted histological assessments using H&E and Masson staining. The regeneration of skin tissue from the wounds collected on day 11 was examined to explore the mechanism of rapid healing observed in the AgNPs-PMT group (Figure 6A). H&E staining showed that the wound surface in the AgNPs-PMT group exhibited a nearly intact epithelial structure with abundant collagen fibers. In contrast, the number of inflammatory cells was significantly reduced compared to the control group. Masson staining revealed substantial collagen deposition in the AgNPs-PMT-treated wounds, indicating that AgNPs-PMT could accelerate wound healing by promoting collagen deposition. Additionally, the presence of some skin appendages, such as hair follicles, was found in the AgNPs-PMT group, suggesting that it not only promoted wound healing but also contributed to the regeneration of skin appendages after healing.

Diabetic infected wounds are often hindered in their healing due to the high-glucose microenvironment, which suppresses angiogenesis. A significant amount of angiogenesis is crucial to supply oxygen, nutrients, and growth factors to the wound area, thereby promoting healing. To evaluate the ability of AgNPs-PMT to promote wound healing, we further assessed angiogenesis in the treated wounds through immunohistochemical staining for CD31 and VEGF, two key markers of angiogenesis. Immunohistochemical images revealed that the AgNPs-PMT group had a higher number of positively stained brown cells for both CD31 and VEGF, indicating upregulation of CD31 and VEGF levels at the wound site (Figure 6B–D). In summary, these data suggest that AgNPs-PMT can clear the bacteria in the infected wound area, alleviate inflammation, and promote the formation of new blood vessels, thereby accelerating wound healing in diabetic mice.

### 3.6. Biosafety Assay

**Figure 7:**
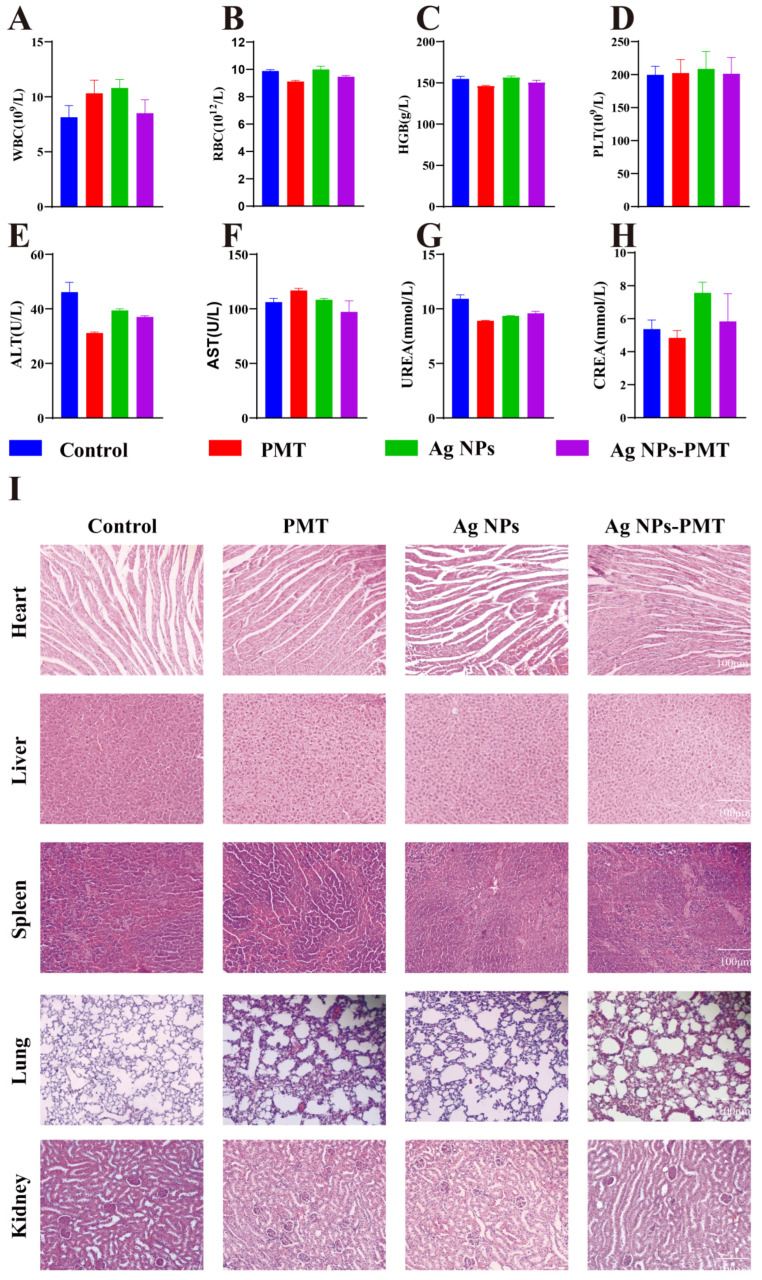
Biosafety assay of AgNPs-PMT in diabetic mice. (**A**–**D**) Blood tests of white blood cell count (WBC), red blood count (RBC), hemoglobin (HGB), and platelets (PLT). (**E**–**H**) Blood tests of alanine ransaminase (ALT), aspartate transferase (AST), UREA and creatinine (CREA). (**I**) Pathological slices of main internal organs with H&E staining. (n = 3, * *p* < 0.05; ** *p* < 0.01; *** *p* < 0.001, **** *p* < 0.0001).

To assess the biosafety of AgNPs and AgNPs-PMT, blood samples and major organs (heart, liver, spleen, lungs, kidneys) were collected from C57BL/6 mice at the end of the in vivo experiment on day 11. Blood parameters, including white blood cells (WBC), red blood cells (RBC), hemoglobin (HGB), and platelets (PLT), were all within the normal range according to routine blood tests (Figure 7A–D). Biochemical analysis of blood revealed no significant abnormalities or damage to liver (ALB, AST/ALT) and kidney (UREA, CREA) functions (Figure 7E–H). Histological examination using H&E staining showed no pathological abnormalities or inflammatory cell infiltration in any of the major organs, indicating that AgNPs-PMT does not cause noticeable damage to the primary organs (Figure 7I).

In summary, AgNPs-PMT exhibited high biosafety and can be considered a safe treatment for combating antibiotic-resistant bacterial infections and promoting diabetic wound healing.

## 4. Conclusions

In this study, we developed a composite antibacterial hydrogel by incorporating AgNPs into PMT hydrogel. In vitro experiments demonstrated that the AgNPs-PMT composite exhibited superior antibacterial activity through the sustained release of AgNPs compared to either PMT or AgNPs alone. Furthermore, the PMT hydrogel matrix effectively mitigated the cytotoxicity associated with AgNPs, as evidenced by reduced cellular damage in viability assays. In vivo experiments using a model of wound infection in diabetic mice showed that, among the AgNPs-PMT treatment groups, wounds healed the fastest, had the fewest inflammatory cells, and had the highest density of collagen fibrils. These findings collectively indicated that AgNPs-PMT eradicates bacterial colonization, modulates the wound microenvironment, and alleviates inflammatory responses. In conclusion, this study provides a promising strategy for managing drug-resistant infections in chronic wounds.

## Data Availability

All data generated or analyzed during this study are included in this published article and Supporting Information File.

## Supplementary Materials

The following supporting information can be downloaded at: https://www.mdpi.com/article/10.3390/bioengineering13010037/s1, Figure S1: Stability analysis of Ag NPs. The dynamic light scattering (DLS) (A), PDI (B) and Zeta potentials (C) of AgNPs stored at 37 °C.; Figure S2: Stability analysis of PMT stored at 37 °C.; Figure S3: EDS elemental mapping of PMT.; Figure S4: EDS elemental mapping of Ag NPs-PMT.; Figure S5: Gelation time of PMT and Ag NPs-PMT.; Figure S6: Ag^+^ release profile from Ag NPs-PMT at 37 °C.; Figure S7: SEM images of (A)PMT and (B)AgNPs-PMT after having co-cultured with MRSA.; Figure S8: Blood glucose values of mice injected with STZ for five consecutive days.
