# Supplementary material for: Bioengineered Premna Microphylla-Silver Nanoparticle Hydrogel for Multidrug-Resistant Wound Management in Diabetic Therapeutics
Source: Bioengineering (Basel). 2025 Dec 29;13(1):37. doi: 10.3390/bioengineering13010037 (PMC12837174; doi:10.3390/bioengineering13010037)
Supplement: Supplementary file 1 [file bioengineering-13-00037-s001.zip › bioengineering-4027222-supplementary.pdf]

bioengineering

Article

Bioengineered Premna Microphylla-Silver Nanoparticle Hydrogel for Multidrug-Resistant Wound Management in Diabetic Therapeutics

Pengxiang Xu [1,†], Yilong Li [1,2,†], Aidi Tong [1], Zhou Wu [1], Chunyi Tong [1,*] and Bin Liu [1,*]

[1] College of Biology, Hunan Province Key Laboratory of Plant Functional Genomics and Developmental Regulation, Hunan University, Changsha 410082, China; xpx123@hnu.edu.cn (P.X.); li_yilong@peplib.com (Y.L.); tad7223@hnu.edu.cn (A.T.); wuzhou1@hnu.edu.cn (Z.W.)
[2] Zonsen PepLib Biotech Inc., Shifeng District, Zhuzhou 412000, China
* Correspondence: sw_tcy@hnu.edu.cn (C.T.); binliu2001@hotmail.com (B.L.)
† These authors contributed equally to this work.

Supplementary Materials

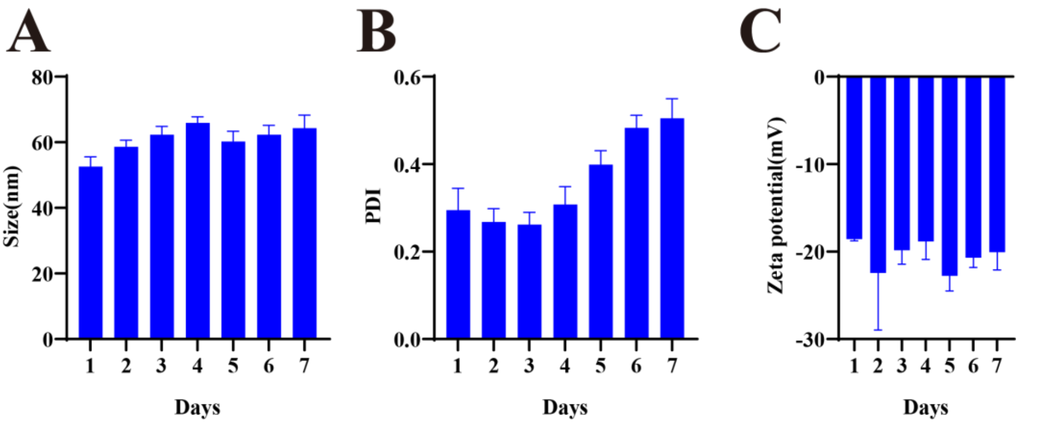

Figure S1. Stability analysis of Ag NPs. The dynamic light scattering (DLS) (A), PDI(B) and Zeta potentials(C) of AgNPs stored at 37 °C.

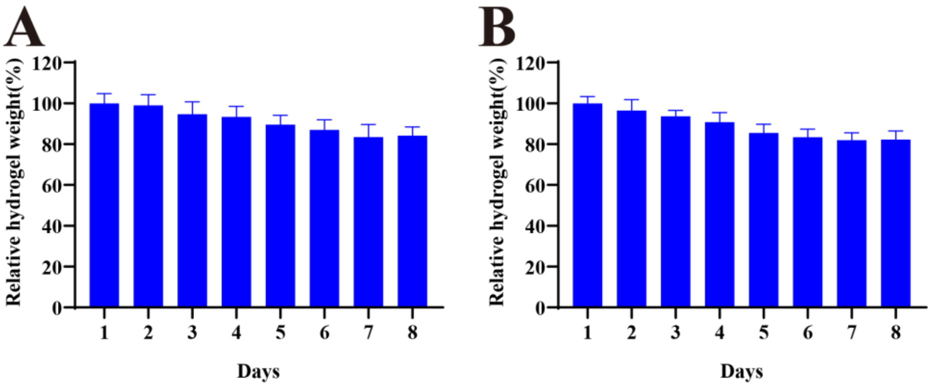

Figure S2 Stability analysis of PMT stored at 37 °C.

 https://doi.org/10.3390bioengineering13010037

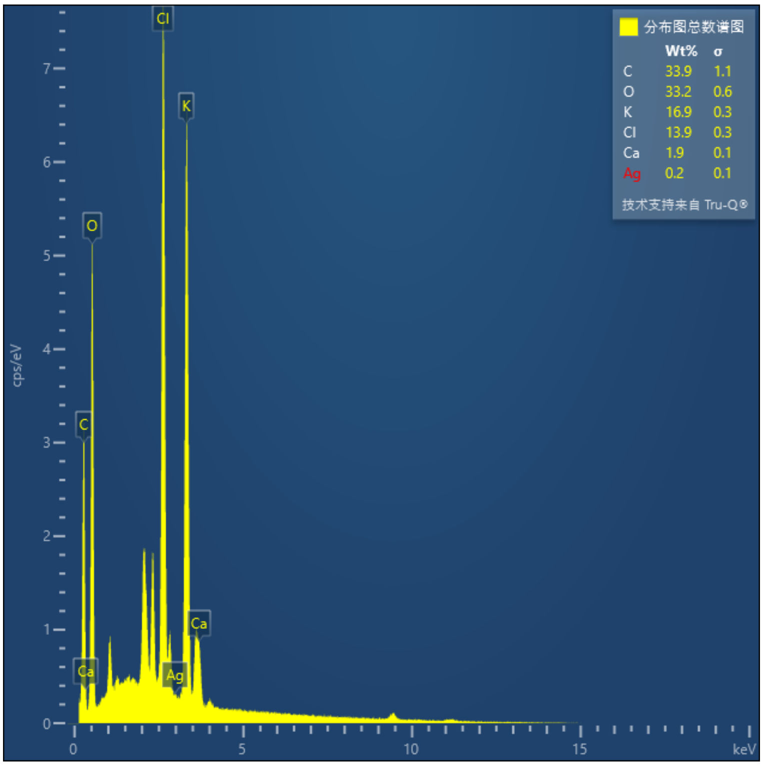

Figure S3 EDS elemental mapping of PMT.

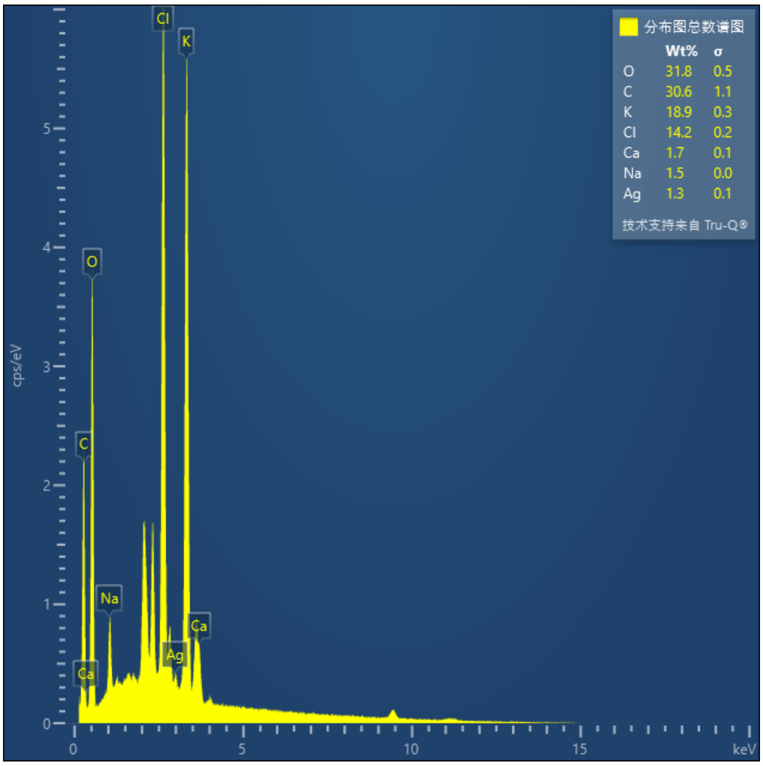

Figure S4 EDS elemental mapping of Ag NPs-PMT.

https://doi.org/10.3390bioengineering13010037

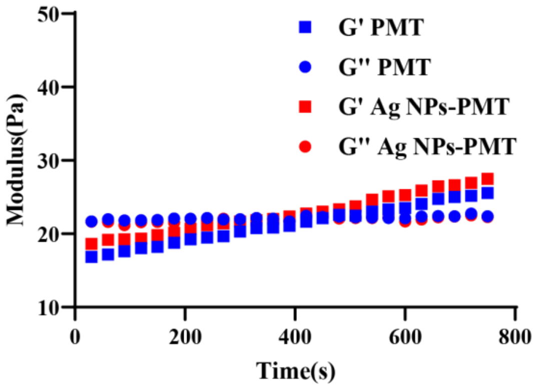

Figure S5. Gelation time of PMT and Ag NPs-PMT.

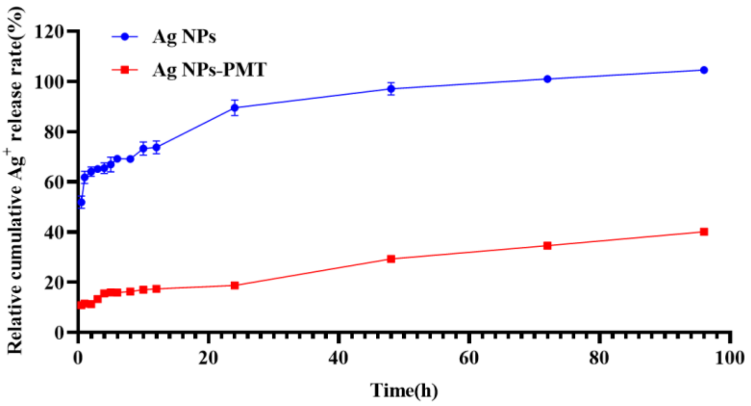

Figure S6. Ag^{+} release profile from Ag NPs-PMT at 37 °C.

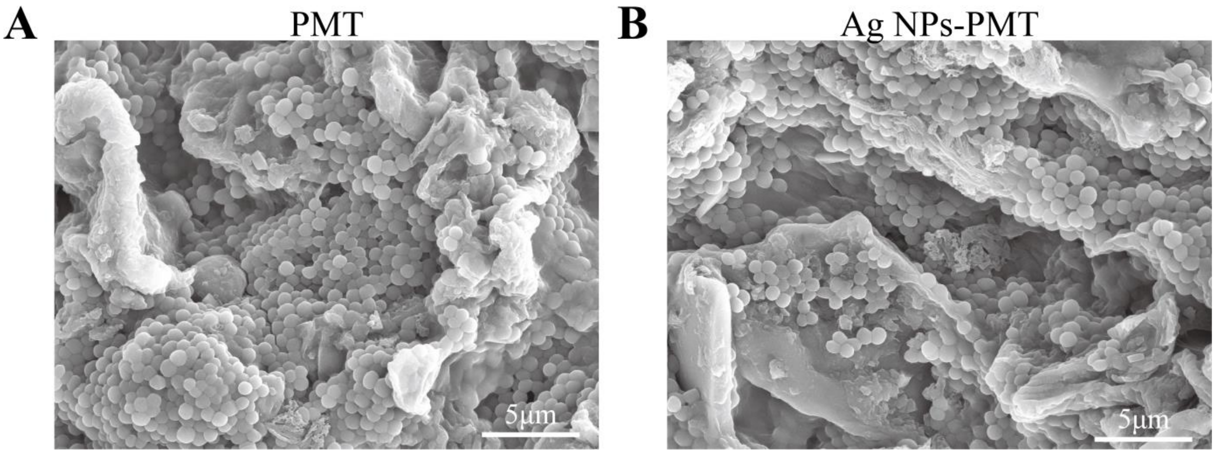

Figure S7. SEM images of (A)PMT and (B)AgNPs-PMT after having co-cultured with MRSA.

https://doi.org/10.3390bioengineering13010037

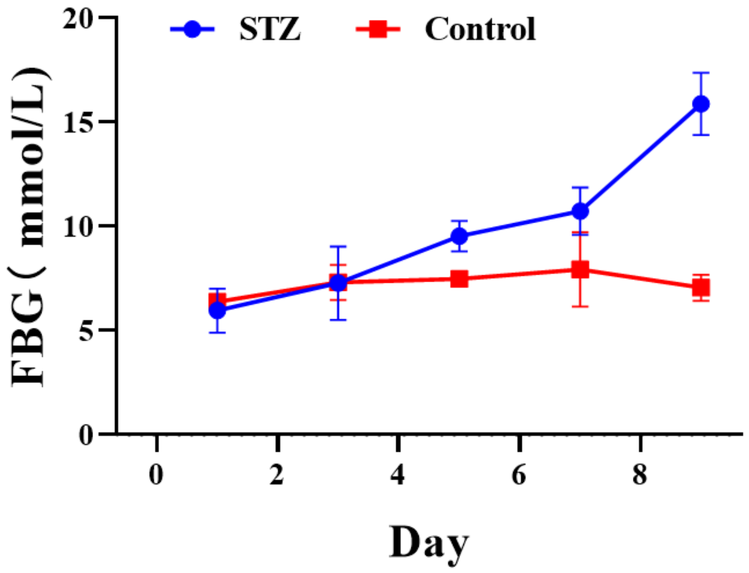

Figure S8. Blood glucose values of mice injected with STZ for five consecutive days.

Disclaimer/Publisher's Note: The statements, opinions and data contained in all publications are solely those of the individual author(s) and contributor(s) and not of MDPI and/or the editor(s). MDPI and/or the editor(s) disclaim responsibility for any injury to people or property resulting from any ideas, methods, instructions or products referred to in the content.

https://doi.org/10.3390bioengineering13010037
